# *ACE2* expression in adipose tissue is associated with cardio-metabolic risk factors and cell type composition—implications for COVID-19

**DOI:** 10.1038/s41366-022-01136-w

**Published:** 2022-05-20

**Authors:** Julia S. El-Sayed Moustafa, Anne U. Jackson, Sarah M. Brotman, Li Guan, Sergio Villicaña, Amy L. Roberts, Antonino Zito, Lori Bonnycastle, Michael R. Erdos, Narisu Narisu, Heather M. Stringham, Ryan Welch, Tingfen Yan, Timo Lakka, Stephen Parker, Jaakko Tuomilehto, Jeffrey Seow, Carl Graham, Isabella Huettner, Sam Acors, Neophytos Kouphou, Samuel Wadge, Emma L. Duncan, Claire J. Steves, Katie J. Doores, Michael H. Malim, Francis S. Collins, Päivi Pajukanta, Michael Boehnke, Heikki A. Koistinen, Markku Laakso, Mario Falchi, Jordana T. Bell, Laura J. Scott, Karen L. Mohlke, Kerrin S. Small

**Affiliations:** 1 Department of Twin Research and Genetic Epidemiology, King’s College London, London, UK; 2 Department of Biostatistics and Center for Statistical Genetics, School of Public Health, University of Michigan, Ann Arbor, MI USA; 3 Department of Genetics, University of North Carolina, Chapel Hill, NC USA; 4 Department of Computational Medicine & Bioinformatics, University of Michigan, Ann Arbor, MI USA; 5 Department of Molecular Biology, Massachusetts General Hospital, Boston, MA 02114 USA; 6 Department of Genetics, Harvard Medical School, Boston, MA 02114 USA; 7 National Human Genome Research Institute, National Institutes of Health, Bethesda, MD USA; 8 Institute of Biomedicine/Physiology, University of Eastern Finland, Kuopio, Finland; 9 Kuopio Research Institute of Exercise Medicine, Kuopio, Finland; 10 Department of Clinical Physiology and Nuclear Medicine, Kuopio University Hospital, University of Eastern Finland, Kuopio, Finland; 11 University of Helsinki and Department of Medicine, Helsinki University Hospital, Helsinki, Finland; 12 Department of Public Health, University of Helsinki, Helsinki, Finland; 13 Diabetes Research Group, King Abdulaziz University, Jeddah, Saudi Arabia; 14 Department of Infectious Diseases, School of Immunology & Microbial Sciences, King’s College London, London, UK; 15 Department of Human Genetics and Institute for Precision Health, David Geffen School of Medicine at UCLA, Los Angeles, CA USA; 16 Department of Public Health and Welfare, Finnish Institute for Health and Welfare, Helsinki, Finland; 17 Minerva Foundation Institute for Medical Research, Helsinki, Finland; 18 Department of Medicine, University of Eastern Finland, Kuopio, Finland; 19 Kuopio University Hospital, Kuopio, Finland

**Keywords:** Risk factors, Genetics, Obesity, Type 2 diabetes

## Abstract

**Background:**

COVID-19 severity varies widely. Although some demographic and cardio-metabolic factors, including age and obesity, are associated with increasing risk of severe illness, the underlying mechanism(s) are uncertain.

**Subjects/methods:**

In a meta-analysis of three independent studies of 1471 participants in total, we investigated phenotypic and genetic factors associated with subcutaneous adipose tissue expression of *Angiotensin I Converting Enzyme 2* (*ACE2*), measured by RNA-Seq, which acts as a receptor for SARS-CoV-2 cellular entry.

**Results:**

Lower adipose tissue *ACE2* expression was associated with multiple adverse cardio-metabolic health indices, including type 2 diabetes (T2D) (*P* = 9.14 × 10^−6^), obesity status (*P* = 4.81 × 10^−5^), higher serum fasting insulin (*P* = 5.32 × 10^−4^), BMI (*P* = 3.94 × 10^−4^), and lower serum HDL levels (*P* = 1.92 × 10^−7^). *ACE2* expression was also associated with estimated proportions of cell types in adipose tissue: lower expression was associated with a lower proportion of microvascular endothelial cells (*P* = 4.25 × 10^−4^) and higher proportion of macrophages (*P* = 2.74 × 10^−5^). Despite an estimated heritability of 32%, we did not identify any proximal or distal expression quantitative trait loci (eQTLs) associated with adipose tissue *ACE2* expression.

**Conclusions:**

Our results demonstrate that individuals with cardio-metabolic features known to increase risk of severe COVID-19 have lower background *ACE2* levels in this highly relevant tissue. Reduced adipose tissue *ACE2* expression may contribute to the pathophysiology of cardio-metabolic diseases, as well as the associated increased risk of severe COVID-19.

## Introduction

Since December 2019 the COVID-19 pandemic has swept the world, with over 500 million confirmed cases worldwide and more than six million fatalities reported at the time of writing [1]. A distinctive and challenging aspect of COVID-19 is its wide disease spectrum [2]. While initial reports focused on respiratory symptoms [3, 4], it is now clear that severe COVID-19 also affects the cardiovascular and renal systems substantially [5]. Marked differences in clinical outcomes have been associated with demographic features including age and sex, with older age and male sex having been associated with increased risk of severe COVID-19, as have comorbidities including cardiovascular disease, diabetes, and obesity [2, 4, 5].

ACE2 has become central in understanding COVID-19 pathogenesis, as SARS-CoV-2 employs ACE2 as a receptor for cellular entry [6, 7]. *ACE2* is expressed in a number of tissues and cell types [6, 8–11]. It is currently unclear whether higher ACE2 levels are harmful or beneficial in the context of COVID-19. On the one hand, higher levels of ACE2 may provide additional targets enabling viral invasion of *ACE2*-expressing cells; on the other, higher ACE2 has beneficial effects in regulation of the renin-angiotensin system (RAS), through controlling hypertension and associated cardio-metabolic disorders [12–14].

Obesity, defined as a body mass index (BMI) above 30 kg/m^2^ [15], is one of the strongest reported risk factors for severe COVID-19 [2, 5], but the mechanism underlying this remains unclear. In the Genotype-Tissue Expression (GTEx) resource, capturing gene expression in 54 tissues, adipose tissue ranks among the highest sites of *ACE2* expression in the body [11, 16] (Supplementary Fig. 1). Several potential mechanisms have been proposed for the contribution of adiposity to COVID-19 severity [17–21], which may be multi-factorial. Critically, *ACE2* in adipose tissue is key in balancing local adipose RAS, disruption of which may then lead to wider systemic RAS effects [18]. Adipose tissue is also a prime candidate tissue contributor to the widely-reported cytokine storm characteristic of severe COVID-19 [17, 18]. Most importantly, obesity is a potentially modifiable risk factor for COVID-19 severity. Thus, deeper understanding of the contribution of adipose tissue to COVID-19 severity, and to cardio-metabolic risk more generally, may inform better therapeutic strategies, and motivate policies supporting healthy weight achievement and maintenance programmes.

Given the dual relevance of ACE2, in maintenance of cardio-metabolic health generally and as SARS-CoV-2 receptor specifically, it is of interest to explore whether adipose tissue *ACE2* expression is associated with specific demographic and phenotypic traits also associated with COVID-19 severity. In this study, we therefore investigated phenotypic and genetic factors associated with *ACE2* gene expression in subcutaneous adipose tissue from a total of 1,471 participants drawn from three cohorts [22–24], representing the largest meta-analysis of adipose tissue gene expression studies to date.

## Materials and methods

### Sample collection

#### TwinsUK

Gene expression data, measured by RNA-Seq, was available for up to 804 female twins from the TwinsUK cohort [25]. Sample collection and processing in TwinsUK has been reported fully elsewhere [25]. Briefly, punch biopsies were taken from a sun-protected area of the abdomen from each participant. Subcutaneous adipose tissue and skin were separated and RNA extracted for each tissue type. Lymphoblastoid cell lines (LCL) were generated from blood samples collected at the time of biopsy. Gene expression in adipose tissue (*n* = 765), skin (*n* = 706), LCLs (*n* = 804), and whole blood (*n* = 389) was measured by RNA sequencing, as previously described [25].

#### METSIM

Sample collection and processing has previously been reported for the population-based METSIM cohort, composed of 10,197 males of Finnish ancestry [26]. RNA-sequencing of subcutaneous adipose tissue for 426 male participants collected near the umbilicus by needle biopsy was generated as previously described [23].

#### FUSION

331 living FUSION participants were recruited from Helsinki, Savitaipale, and Kuopio, as previously described [24]. Adipose tissue biopsies were obtained and processed from 296 of the 331 participants following the same general protocol as previously described for the concurrently-obtained muscle biopsies [24]. Biopsies were taken under local anaesthetic, by surgical scalpel from abdominal subcutaneous fat 5–10 cm lateral of the umbilicus, cleaned of blood and other visible non-adipose tissue, and rinsed with 0.9% sodium chloride. Adipose RNA-sequencing was performed as described for muscle RNA-sequencing [24].

### RNA-Seq data processing

#### TwinsUK and METSIM

RNA-Seq reads were aligned to the hg19 reference genome using STAR [27] version 2.4.0.1 (TwinsUK) and 2.4.2a (METSIM), and quality control conducted as previously described [23, 28]. Gene-level counts were calculated using the quan function from QTLtools [29] and Gencode version 19 [30].

#### FUSION

RNA-Seq reads were aligned to the hg19 reference genome using procedures as described previously [24]. Six outlier samples were excluded based on low read coverage, and five outliers based on gene diversity. The allelic RNA-Seq read count distribution was compared to single nucleotide polymorphism (SNP) data from genome-wide association study (GWAS) panels available for the same subjects using verifyBamID [31]; two contaminated samples were identified and removed, as well as one pair of sample swaps corrected. Reported sex of the remaining samples was verified by matching the sex inferred using expression of *XIST* - encoded on the X chromosome and expressed only in cells with a minimum of two X chromosomes—and the mean Y chromosome gene expression. Linear regression of gene expression as a function of age, sex, batch, and RNA integrity number (RIN)—a measure of RNA quality—was performed, in addition to performing principal component analysis (PCA) on the gene expression residuals. The minimum number of PCs were selected to explain 20% of the variance in gene expression. Finally, one non-Finnish subject and one from each of two first-degree relative pairs were removed. A total of 280 samples were retained in the analysis.

### Gene inclusion and normalization

For all three studies, RNA-Seq gene expression data were filtered to retain only genes with 5 or more counts per million (CPMs) in greater than or equal to 25% of individuals in each study. Trimmed mean of M-values between-sample normalisation was applied to gene counts [32], and TMM-normalised gene CPMs were then inverse-normalised prior to all downstream analyses (inverse-normalized *ACE2*).

### Estimation of adipose tissue cell type proportions

As adipose tissue comprises adipocytes, immune cells, and vascular endothelial cells, all of which may be present in differing proportions between samples, it is critical to account for between-sample differences in the composition of cell types within biopsies. Adipose tissue cell type proportions were estimated from RNA-Seq data in TwinsUK using CIBERSORT [33], as previously described [34]. Estimated cell types included in our analysis were adipocytes, microvascular endothelial cells (MVEC) and macrophages.

To estimate the proportions of adipose and blood cell types in the adipose biopsies from the FUSION and METSIM studies, a reference transcriptome was created using RNA-Seq of whole blood (GEO accession GSE67488 [35]), and of four types of purified adipose cells (adipocytes, macrophages, CD4 + T cells, and microvascular endothelial cells) described in Glastonbury et al. [34]. For the FUSION study, reference transcriptome reads were aligned to the hg19 reference genome using the same read mapping and quality control procedure as used for the FUSION adipose RNA-Seq data [24]. Blood/cell-type proportions for each FUSION adipose sample were estimated using the unmix function from DESeq2 v1.18.1 [36]. A gene expression signature matrix was constructed and blood/cell-type proportions were estimated for each METSIM adipose sample using CIBERSORT [33].

### Phenotypic association analyses

For quantitative phenotypic trait association analyses, in TwinsUK we excluded a subset of participants who had not fasted on the day of biopsy and for whom biochemical measurements were not matched to the day of biopsy, or for whom fasting and time of visit covariates used in our analyses were not available (*n* = 161). Participants with T2D were excluded from all analyses in all studies except those modelling association of diabetes status with *ACE2* expression.

We assessed association between *ACE2* gene expression levels (Gencode ID ENSG00000130234.6) and phenotypic traits including age, BMI, serum fasting insulin, fasting plasma glucose, serum HDL and LDL cholesterol, total triglycerides, systolic and diastolic blood pressure, obesity, T2D status (TwinsUK only) and sex (FUSION only). For obesity status, cases (TwinsUK: *n* = 119; METSIM: *n* = 67; FUSION: *n* = 68) were defined as participants with BMI ≥30 kg/m^2^, while controls (TwinsUK: *n* = 259; METSIM: *n* = 140; FUSION: *n* = 79) were defined as participants with BMI < 25 kg/m^2^. In TwinsUK, established T2D case status (*n* = 31) was defined through longitudinal patient self-reports, and longitudinal records of elevated fasting plasma glucose (≥7 mmol/l) and serum fasting insulin levels. In this study, individuals were defined as T2D controls (*n* = 567) if they had no longitudinal records of elevated fasting plasma glucose nor serum fasting insulin, as well as no self-reports of diabetes. Individuals with insulin resistance, defined as participants with longitudinally-elevated serum fasting insulin levels (>60 pmol/l) with fasting plasma glucose levels consistently below 6.0 mmol/l (*n* = 130) were excluded from T2D association analyses. In FUSION, study participants underwent a 2-h, four-point oral glucose tolerance test (OGTT) after a 12-h overnight fast, and T2D, impaired fasting glucose (IFG), impaired glucose tolerance (IGT) and normal glucose tolerance (NGT) status was assigned per WHO criteria [37]. We also assessed association of adipose tissue *ACE2* expression with use of ACE inhibitors or angiotensin II receptor blockers, as well as adipose tissue estimated cell type proportions, specifically each of microvascular endothelial cell, macrophage and adipocyte proportions, estimated as described above.

We performed rank-based inverse normal transformation of all quantitative phenotypic traits. Association between *ACE2* gene expression levels and each phenotypic trait was assessed by fitting linear mixed effects models using the lmer function from the lme4 package [38] (TwinsUK) or linear regression models using the lm function (METSIM and FUSION) in R [39] version 3.5.1 (TwinsUK and METSIM) or version 3.6.3 (FUSION). Inverse normalised *ACE2* expression was treated as a continuous dependent variable, and each phenotypic trait in turn was treated as an independent fixed effect.

Covariates included in the TwinsUK study were: age, BMI (for all phenotypes except BMI and obesity), number of hours fasted, and the RNA-Seq technical covariates sample median transcript integrity number (TIN—a measure of sample quality) [40], median insert size, and mean GC content included as fixed effects. Random effects included were date of RNA sequencing, morning *versus* afternoon visit time, RNA extraction batch, RNA-Seq primer index, family and zygosity. Family and zygosity are both random effects that describe the family a twin belongs to, and their clonality (MZ/DZ status). Each family identifier is specific to a twin pair, and each zygosity label is identical for each pair of MZ twins, while individual DZ co-twins are each given a unique zygosity label. In TwinsUK, the full model was then compared to a null model where the phenotypic trait of interest was omitted, using a 1 degree of freedom ANOVA.

Covariates included in the METSIM study were: age, BMI (for all phenotypes except BMI and obesity), blood cell type proportion, and RNA-Seq technical covariates read deletion size, mean read insertion size, TIN, and sequencing batch.

Covariates included in the FUSION study were: age, sex, BMI (for all phenotypes except BMI and obesity), collection site, blood cell type proportion, four genetic PCs, and RNA-Seq technical covariates mean GC content, median read insertion size, RNA integrity number (RIN), TIN, and sequencing batch included as fixed effects. In the FUSION study, which included both males and females, analyses were also performed in males and females separately, excluding the sex covariate in the models.

Sensitivity analyses assessing the impact of adjusting for body fat distribution as opposed to BMI were conducted in all three studies. In TwinsUK, dual-energy X-ray absorptiometry (DEXA) scans were used to quantify fat volume in android and gynoid regions following standard manufacturer’s recommendations (DXA; Hologic QDR 4500 plus), from which android/gynoid ratio was calculated. In each of the METSIM and FUSION studies, waist/hip ratio (WHR) was used as a measure of body fat distribution. Sensitivity analyses were run by including either android/gynoid ratio (TwinsUK) or WHR (METSIM and FUSION) as a covariate instead of BMI in the models above. Analyses in the FUSION study were conducted in males and females separately.

Sensitivity analyses were conducted for all three studies, including estimated MVEC cell type proportion, then both estimated MVEC and macrophage cell type proportions, as an additional covariate(s) in the models above.

### Meta-analysis of phenotypic associations with adipose tissue *ACE2* expression

Given the demographic differences between our study samples, we selected a meta-analysis approach that permited the true effect to vary between studies. We thus conducted a random-effects inverse-variance weighted meta-analysis of up to 1,237 participants, combining *ACE2* gene expression-phenotype association results from the TwinsUK, METSIM and FUSION studies (See Supplementary Tables 1–6 for trait-specific numbers of participants). Meta-analysis was conducted for 13 out of 15 traits using the rma function (restricted maximum likelihood method) from the metafor package [41] in R version 3.5.1 [39]. Association of T2D with *ACE2* was assessed in TwinsUK only, as it was the only study with participants with established T2D at time of biopsy. Association of *ACE2* expression with sex was assessed in FUSION only, given that the TwinsUK and METSIM studies included only females or males, respectively, to avoid biasing of estimates that may result from confounding by study-specific technical differences.

In order to take into account the number of tests conducted, a multiple testing correction threshold of *P* < 3.33 × 10^−3^, calculated using a Bonferroni correction for 15 traits (0.05/15), was used to assess significance of phenotypic associations with *ACE2* expression.

### Detection of COVID-19 antibodies in TwinsUK participants

#### Enzyme linked immunosorbent assay (ELISA)

Presence of immunoglobulin G (IgG) to SARS-CoV-2 nucleoprotein (N) and spike (S) in serum from TwinsUK participants was measured using ELISA as previously described [42, 43]. Serum samples were diluted to 1:50. Optical density (OD) values >4-fold above assay background was used as a cut-off for seropositivity [42].

#### Lateral flow assays

Fortress COVID-19 Total Ab Device (Fortress Diagnostics Ltd) was used for qualitative detection of total antibodies against SARS-CoV-2, employing a chromatographic lateral flow device within a cassette format. In total, 10 μl of serum was added to the specimen window immediately followed by two drops of diluent buffer to the buffer window. Each cassette was left for exactly 10 min before being read by a trained person. Seropositivity was indicated by the presence of a visible red line in the Test Zone and results were classified as either IgG positive only, IgM positive only, IgG & IgM positive, negative or invalid. Prior to and during the cassette readings the personnel responsible for readings undertook a verification process looking at inter-rater and intra-rater reading variability.

### Identification of SARS-CoV-2 seropositive individuals in TwinsUK

The presence of SARS-CoV-2 antibodies was assessed in 459 TwinsUK subjects for whom adipose tissue gene expression data were available, using either ELISA or lateral flow SARS-CoV-2 antibody tests as described. Individuals were classified as SARS-CoV-2 seropositive if they received a positive antibody test result from either method.

### Longitudinal COVID-19 symptom reporting in TwinsUK

Experience of potential COVID-19 symptoms during the period between February-July 2020 was collected from TwinsUK participants using detailed postal health questionnaires. The symptoms which the participants were asked to report on are shown in Supplementary Table 7.

### Association of adipose tissue *ACE2* expression with COVID-19 symptom presentation in SARS-CoV-2 seropositive participants from TwinsUK

We assessed association between adipose tissue *ACE2* gene expression levels and presentation of one or more core COVID-19 symptoms (fever, persistent cough, anosmia) and/or one or more of the more severe COVID-19 symptoms (shortness of breath, chest pain or chest tightness) in a sample of 32 unrelated subjects, without diabetes, from TwinsUK who were found to be seropositive for SARS-CoV-2 antibodies, and for whom COVID-19 symptom reports were available.

Association was assessed by fitting a logistic regression model using the glm function in R version 3.5.1. The presence or absence of core and/or severe COVID-19 symptoms was treated as a binary dependent variable; *ACE2* expression, adjusted for RNA-Seq technical covariates (sample median transcript integrity number (TIN), median insert size, mean GC content, date of sequencing and primer index) as well as age and BMI at time of biopsy, was treated as an independent fixed effect, with BMI and age at antibody assessment included as covariates in the model.

### Correlation of phenotypic traits

The correlations between phenotypic traits were assessed in TwinsUK, which was the largest study included in our analyses. Phenotypic trait correlations were assessed using a Spearman correlation in unrelated participants from TwinsUK (*n* = 441), in R version 3.5.1 [39].

## Results

We quantified adipose *ACE2* expression levels in bulk RNA-Seq data from TwinsUK [22, 44, 45] (765 female mono- and dizygotic twins), 426 males from the Metabolic Syndrome in Men (METSIM) study [23], and 149 Finnish males and 131 Finnish females from the Finland-United States Investigation of NIDDM Genetics (FUSION) Tissue Biopsy Study [24]. The cohorts differ in country of origin, male:female composition, age distribution, and underlying ascertainment criteria (Table 1). Study participants ranged in age from 35 to 85 years, with variation between studies (Table 1 and Supplementary Fig. 2). While TwinsUK and METSIM are representative of the UK [22] and Kuopio Finland [23] populations respectively, the FUSION study design was enriched for individuals with abnormal glycaemic indices at time of biopsy.

**Table 1:** Descriptive statistics of study participants. Quantitative traits are reported as median [1st–3rd quartiles].

	TwinsUK	METSIM	FUSION
N [% female]	765 [100]	426 [0]	280 [47]
Age (years)	59 [52–65]	54 [51–59]	60 [55–65]
BMI (kg/m^2^)	25.6 [23.3–28.8]	26.2 [24.6–28.6]	26.7 [24.2–28.8]
Serum fasting insulin (pmol/L)	38.0 [25.0–59.5]	35.4 [25.2–55.8]	43.8 [28.8–59.1]
Fasting plasma glucose (mmol/L)	4.9 [4.6–5.2]	5.7 [5.4–6.0]	5.9 [5.6–6.3]
Serum HDL (mmol/L)	1.80 [1.53–2.14]	1.45 [1.20–1.70]	1.44 [1.20–1.70]
Serum LDL (mmol/L)	3.18 [2.57–3.82]	3.43 [2.96–3.96]	3.34 [2.78–3.89]
Serum triglycerides (mmol/l)	0.96 [0.72–1.33]	1.17 [0.88–1.62]	1.17 [0.89–1.56]
Systolic BP (mmHg)	128 [118–139]	132 [123–141]	133 [121–145]
Diastolic BP (mmHg)	78 [71–85]	87 [81–93]	82 [76–87]
Adipose tissue *ACE2* expression (TMM-adjusted CPMs)	1.86 [1.16–3.05]	1.25 [0.82–1.83]	1.15 [1.07–2.62]

Statistics for all traits are calculated including only participants without diabetes. *ACE2* expression levels are reported as trimmed mean of M-values (TMM)-adjusted counts per million (CPMs).

We hypothesised that the true effect sizes of *ACE2* gene expression-phenotypic associations may vary between cohorts that differ in their demographic characteristics, and due to technical and methodological differences between the studies. We therefore assessed association of *ACE2* gene expression with demographic and phenotypic traits in each of the TwinsUK, METSIM and FUSION studies, adjusting for age and BMI, and then combined the association results from all three studies by conducting a random-effect inverse-variance-weighted meta-analysis. Results of meta-analysis of the three studies showed consistency in association signals across cohorts for some traits, but heterogeneity for others.

**Fig. 1:**
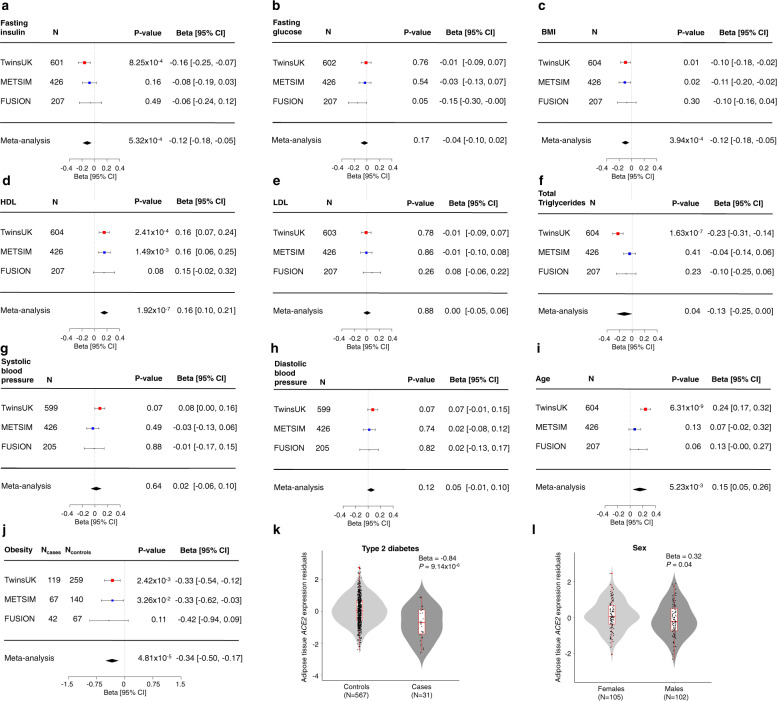
Associations of adipose tissue *ACE2* expression levels with cardio-metabolic and demographic traits in the TwinsUK, METSIM and FUSION cohorts. Squares with error bars represent the standardised β coefficients and their 95% confidence intervals for association of *ACE2* expression levels with each trait (derived from linear/linear mixed effects regression models), with meta-analysis effect sizes and 95% confidence intervals (random-effects meta-analysis) shown as black diamonds. N represents the sample size for each analysis. **a** serum fasting insulin, (**b**) fasting plasma glucose, (**c**) BMI (**d**) serum HDL cholesterol, (**e**) serum LDL cholesterol, (**f**) serum total triglycerides, (**g**) systolic blood pressure, (**h**) diastolic blood pressure, (**i**) age, (**j**) obesity status. Association of *ACE2* expression with (**k**) type 2 diabetes status in TwinsUK and (**l**) sex in the FUSION study. Boxplots (**k**, **l**) display the median and inter-quartile range (IQR), with whiskers corresponding to ±1.5*IQR.

Adipose tissue *ACE2* expression was relatively low across all three cohorts (Table 1, Supplementary Figs. 3 and 4). In our meta-analysis, lower adipose tissue *ACE2* expression levels were associated with higher serum fasting insulin (β [95% CI] = −0.12 [−0.18, −0.05]; *P* = 5.32 × 10^−4^), and higher body mass index (BMI) (β [95% CI] = −0.10 [−0.16, −0.04]; *P* = 3.94 × 10^−4^) (Fig. 1 and Supplementary Table 1). Consistent with the association observed for BMI, participants with obesity had lower adipose tissue *ACE2* expression compared to normal-weight controls (β [95% CI] = −0.34[−0.50, −0.17]; *P* = 4.81 × 10^−5^) (Fig. 1 and Supplementary Table 1).

TwinsUK includes a subset of individuals diagnosed with T2D prior to biopsy. Within TwinsUK, T2D status was associated with lower adipose tissue *ACE2* expression compared to normoglycaemic controls (β[95% CI] = −0.84[−1.21, −0.47]; *P* = 9.14 × 10^−6^). No association was observed with fasting glucose in our study (*P* > 0.05) (Fig. 1 and Supplementary Table 1).

Lower *ACE2* expression was strongly associated with lower serum HDL cholesterol (β [95% CI] = 0.16 [0.10, 0.21]; *P* = 1.92 × 10^−7^) (Fig. 1 and Supplementary Table 1). Association of total serum triglycerides with adipose tissue *ACE2* expression showed heterogeneity between studies (heterogeneity indices: I^2^ = 73.24; Q-test *P* = 0.01). Results of meta-analysis of all three studies showed only nominally significant association between lower *ACE2* expression and higher triglyceride levels (β [95% CI] = −0.13[−0.25, 0.00]; *P* = 4.15 × 10^−2^), but this association was not significant after multiple testing correction (MTC threshold *P* < 3.33 × 10^−3^). In TwinsUK, lower *ACE2* expression in adipose tissue was strongly associated with higher total triglyceride levels (β [95% CI] = −0.23 [−0.31, −0.14]; *P* = 1.63 × 10^−7^). Adipose tissue *ACE2* expression was not associated with serum LDL cholesterol, nor with systolic or diastolic blood pressure (*P* > 0.05) (Fig. 1 and Supplementary Table 1).

Severity of COVID-19 is associated with increasing age [2–4, 46]. Upon meta-analysis, higher adipose *ACE2* expression levels were nominally associated with older age (β [95% CI] = 0.15 [0.05, 0.26]; *P* = 5.23 × 10^−3^), but not significant after MTC (Fig. 1 and Supplementary Table 1). However, we observed heterogeneity in age effects between studies (Heterogeneity I^2^ = 71.38; Cochrane’s Q-test *P* = 0.02) potentially driven by differences in the age distribution of the studies. In the study, with the largest age range, TwinsUK (38–85 years), higher adipose tissue *ACE2* expression was strongly associated with older age (β [95% CI] = 0.25[0.17, 0.32]; *P* = 6.31 × 10^−9^). Higher *ACE2* expression in adipose tissue from older individuals was consistent with previous reports of age-dependent *ACE2* expression in nasal epithelium [47]. However, in skin samples from the same TwinsUK participant, lower *ACE2* expression was associated with increased age (β [95% CI] = −0.23[−0.31, −0.14]; *P* = 4.77 × 10^−7^), supporting heterogeneity of effects between tissues [11] (Supplementary Fig. 5). We also noted that adipose tissue gene expression levels of *ACE2* were not associated with use of ACE inhibitors or angiotensin II receptor blockers (*P* > 0.05; Supplementary Table 2).

Located on the X chromosome, *ACE2* has been shown to escape from X inactivation [48]. It is therefore of interest to consider whether *ACE2* expression differences may contribute to the observed differences in COVID-19 severity and clinical outcomes. Association of *ACE2* expression with sex was assessed only in the FUSION study, which included both males and females. Consistent with reports from GTEx [11], *ACE2* expression was nominally higher in females in FUSION (β [95% CI] = 0.32 [0.02, 0.61]; *P* = 0.037), but this difference was not significant after multiple testing correction (Fig. 1 and Supplementary Table 1).

Given the well-documented differences in body fat distribution between males and females, we sought to conduct a sensitivity analysis to assess whether phenotypic associations may be confounded by these differences. We therefore adjusted for available measures of body fat distribution in each of the three studies in place of BMI, adjusting for DEXA-derived android/gynoid ratio in TwinsUK, and waist/hip ratio in each of METSIM and FUSION. Sensitivity analyses in the FUSION study were conducted in males and females separately. Phenotypic associations were robust to these adjustments, and were in some cases strengthened (Supplementary Table 3).

**Fig. 2:**
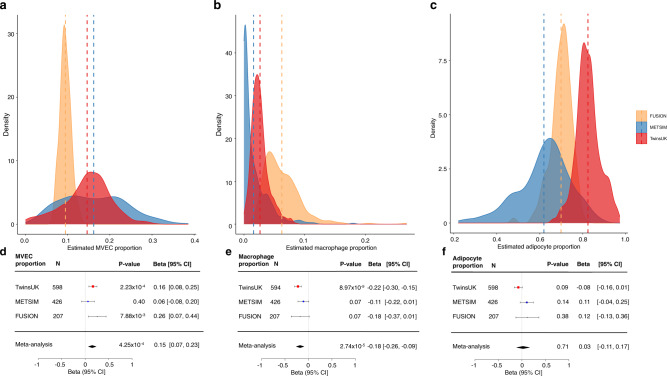
Adipose tissue estimated cell type proportions and their association with *ACE2* expression levels across the TwinsUK, METSIM and FUSION studies. Density plots of estimated cell type proportions of (**a**) microvascular endothelial cells (MVEC), (**b**) macrophages, (**c**) adipocytes in each of the TwinsUK, METSIM, and FUSION studies. Vertical dashed lines correspond to the study mean. Association of adipose tissue *ACE2* expression levels with estimated (**d**) MVEC, (**e**) macrophage and (**f**) adipocyte proportions. Squares with error bars represent the standardised β coefficients and their 95% confidence intervals for association of *ACE2* expression levels with each of the traits (derived from linear/linear mixed effects regression models), with the meta-analysis effect sizes and 95% confidence intervals (random-effects meta-analysis) shown as black diamonds. N represents the sample size for each analysis.

Adipose tissue consists of multiple cell types, gene expression of all of which is represented in bulk RNA-Seq data, and to our knowledge, it is still unclear which adipose cell types are responsible for expression of *ACE2*. We therefore assessed the relationship between *ACE2* expression in subcutaneous adipose tissue and estimated adipose tissue cell type proportions. Consistent with differences in biopsy methods employed, significant heterogeneity in cell type composition was observed between the three studies (Fig. 2). Despite these differences, a higher proportion of microvascular endothelial cells (MVEC) was associated with higher *ACE2* expression (β [95% CI] = 0.15 [0.07, 0.23]; *P* = 4.25 × 10^−4^), suggesting microvascular endothelial cell subpopulations contribute to *ACE2* expression levels in adipose tissue (Fig. 2 and Supplementary Table 4). Conversely, lower *ACE2* expression was associated with higher macrophage proportion (β [95% CI] = −0.18 [−0.26, −0.09]; *P* = 2.74 × 10^−5^) (Fig. 2 and Supplementary Table 4). Increased macrophage infiltration in adipose tissue has been associated with a pro-inflammatory state [49]. *ACE2* expression was not associated with adipocyte proportion (*P* > 0.05; Fig. 2 and Supplementary Table 4).

To establish whether phenotypic associations with *ACE2* expression were simply reflecting differences in adipose tissue cell type composition [34], we conducted sensitivity analyses including the estimated proportion of MVECs as a covariate in our models. While strength of association was attenuated for some phenotypes, the associations were largely robust to adjustment for MVEC proportion (Supplementary Table 5), suggesting phenotypic associations were not driven solely by differences in the proportions of *ACE2*-expressing cell types. Furthermore, correlation patterns between phenotypic traits, including age and each of adipose tissue *ACE2* expression and MVEC proportions, were notably different (Supplementary Fig. 6). In contrast, phenotypic associations were notably attenuated upon inclusion of both MVEC and macrophage proportions as covariates (Supplementary Table 6).

**Fig. 3:**
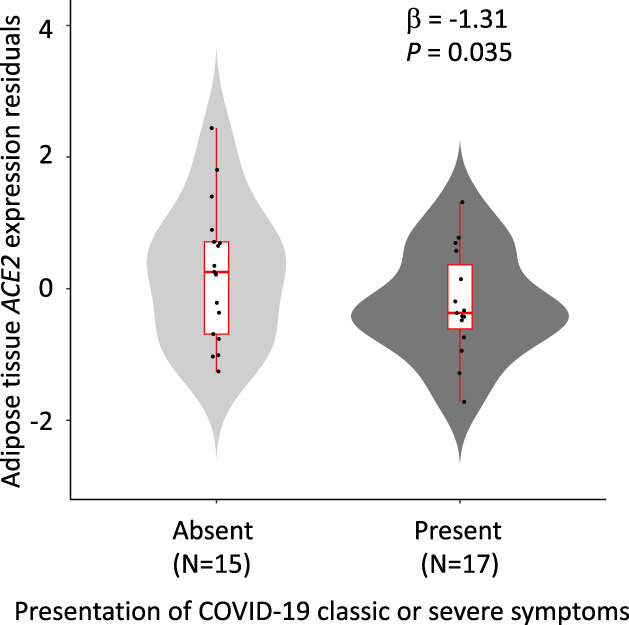
Adipose tissue *ACE2* expression in SARS-CoV-2 seropositive subjects. Adipose tissue *ACE2* expression residuals (adjusted for age, BMI and RNA-Seq technical covariates) are shown in SARS-CoV-2 seropositive TwinsUK participants who reported presenting one or more classic or severe COVID-19 symptoms compared to seropositive individuals who did not display any of the classic or severe COVID-19 symptoms. Boxplots display the median and inter-quartile range (IQR), with whiskers corresponding to ±1.5*IQR. Effect size and *P* value were calculated using a logistic regression model adjusting for current age and BMI.

We next sought to investigate whether background adipose tissue *ACE2* expression levels were associated with COVID-19 clinical presentation in individuals confirmed to have experienced SARS-CoV-2 infection. As part of the TwinsUK COVID-19 research initiative, we assessed SARS-CoV-2 antibody status and COVID-19 symptom reports in TwinsUK participants (Supplementary Table 7), of whom 459 overlapped our adipose tissue gene expression study sample. In unrelated TwinsUK participants seropositive for SARS-CoV-2 antibodies (*N* = 32), we assessed whether background *ACE2* expression was associated with presentation of one or more core COVID-19 symptoms (fever, persistent cough, anosmia) and/or one or more of the more severe COVID-19 symptoms (shortness of breath, chest pain or chest tightness). Adipose tissue *ACE2* expression was nominally lower in SARS-CoV-2 seropositive subjects who exhibited one or more classic or severe symptoms of COVID-19 compared to seropositive subjects who did not exhibit any of these symptoms (β [95% CI] = −1.31 [−2.53, −0.09]; *P* = 0.035; Fig. 3). Further studies in larger sample sizes will be required to confirm these findings.

Finally, despite a heritability of 0.32 [95% CI = 0.14–0.50] in TwinsUK, cis- and trans-eQTL meta-analyses of all three studies did not reveal any significant eQTLs associated with adipose tissue *ACE2* expression (Supplementary Information and Supplementary Fig. 7). Similarly, no proximal methylation probes were associated with genetic variants in cis or trans in the TwinsUK sample (Supplementary Information and Supplementary Fig. 8).

## Discussion

Taken together, our results suggest that lower adipose tissue expression of *ACE2* is associated with multiple adverse cardio-metabolic health indices, all of which are risk factors for severe COVID-19 [2, 4, 5]. Lower adipose tissue *ACE2* expression was associated with diabetes and obesity status, increased serum fasting insulin and triglyceride levels, BMI, and with increased macrophage infiltration in adipose tissue, a marker of inflammation [49]. *ACE2* expression was positively correlated with HDL levels, age and adipose tissue MVEC proportion. Exploratory analyses in a small sample of SARS-CoV-2 seropositive individuals from TwinsUK suggested association of adipose tissue ACE2 expression levels with symptom presentation. While the limited sample size warrants cautious interpretation, these findings highlight the importance of assessing the contribution of adipose tissue *ACE2* expression to COVID-19 severity in larger sample sizes. As previously noted for *ACE2* associations reported in other tissues [50], and given its association with adipose tissue cell type proportions, it remains unclear to what extent these associations are mediated through differences in underlying cell type composition. While sensitivity analyses adjusting for MVEC cell type proportion estimates suggested that the *ACE2* expression-trait associations were robust, estimation of cell type composition from bulk RNA-Seq data in non-blood tissues remains in its infancy. Adipose tissue single-cell RNA sequencing (scRNA-Seq) datasets will be critical to address this question fully. Furthermore, as shown by the opposing directions of effect in skin *versus* adipose tissue for association with age in the TwinsUK sample, *ACE2* expression-trait associations may differ between tissues, supporting the need for caution in their interpretation and extrapolation. The contrast between our observation that adipose tissue *ACE2* levels were positively correlated with age - a strong risk factor for severe COVID-19 - yet negatively correlated with COVID-19 cardio-metabolic risk factors may reflect the multi-systemic nature of COVID-19. There are likely a number of mechanisms that underlie increased risk of severe COVID-19, and these are not necessarily shared by all its demographic and cardio-metabolic risk factors. While adipose tissue may be a key driver of increased susceptibility to severe COVID-19 for some risk factors such as cardio-metabolic risk factors, other tissues and mechanisms may be more important for other traits such as age.

Despite evidence of heritability of adipose tissue *ACE2* expression levels and our meta-analysis sample size, we did not yet identify cis- or trans-eQTLs associated with adipose *ACE2* expression. Increased sample sizes may be needed to detect genetic factors influencing expression of *ACE2* in this important tissue.

Increasing evidence suggests that COVID-19 severity may be modulated by widespread microvascular damage and increased predisposition to thrombotic events [51–53], in which ACE2, and the RAS system more widely, may play a critical role. Adipose tissue is an active endocrine organ which secretes hormones, chemokines and cytokines that contribute to regulation of inflammatory and immune responses [54, 55]. Given the association of *ACE2* expression with cardio-metabolic risk factors for severe COVID-19, it is intriguing to consider whether low levels of adipose *ACE2* expression may contribute to COVID-19 severity in at-risk individuals. SARS-CoV infection reduces ACE2 levels post-infection [56–59]. Our observation that individuals suffering from co-morbidities recognised to be associated with severe COVID-19 may have lower pre-infection ACE2 levels in some tissues may support a theory that pre-existing low ACE2 levels in at risk-individuals are then reduced further upon SARS-CoV-2 infection, contributing to the dysregulation of the RAS characteristic of severe COVID-19 [60]. Further studies will be needed to clarify the contribution of adipose tissue *ACE2* expression levels to COVID-19 severity and beyond.

## Supplementary information

Supplementary Information File

Supplementary Tables 1-8

Supplementary Table 9

Supplementary Table 10

## Data Availability

TwinsUK RNA-Seq data are deposited in the European Genome-phenome Archive (EGA) under accession EGAS00001000805. METSIM RNASeq data have been deposited in the Gene Expression Omnibus (GEO) under accession GSE135134. The dbGaP accession number for the FUSION Tissue Biopsy Study is phs001048.v2.p1.
