# Supplementary material for: ACE2 expression in adipose tissue is associated with cardio-metabolic risk factors and cell type composition—implications for COVID-19
Source: Int J Obes (Lond). 2022 May 20;46(8):1478–86. doi: 10.1038/s41366-022-01136-w (PMC9119844; doi:10.1038/s41366-022-01136-w)
Supplement: Supplementary file 1 — Supplementary Information File [file 41366_2022_1136_MOESM1_ESM.docx]

**SUPPLEMENTARY INFORMATION**

***ACE2* expression in adipose tissue is associated with cardio-metabolic risk factors and cell type composition – implications for COVID-19**

Julia S. El-Sayed Moustafa^1*^, Anne U. Jackson^2*^, Sarah M. Brotman^3*^, Li Guan^4*^, Sergio Villicaña^1^, Amy L. Roberts^1^, Antonino Zito^1,5,6^, Lori Bonnycastle^7^, Michael R. Erdos^7^, Narisu Narisu^7^, Heather M. Stringham^2^, Ryan Welch^2^, Tingfen Yan^7^, Timo Lakka^8,9,10^, Stephen Parker^4^, Jaakko Tuomilehto^11,12,13^, Jeffrey Seow^14^, Carl Graham^14^, Isabella Huettner^14^, Sam Acors^14^, Neophytos Kouphou^14^, Samuel Wadge^1^, Emma L. Duncan^1^, Claire J. Steves^1^, Katie J Doores^14^, Michael H. Malim^14^, Francis S. Collins^7^, Päivi Pajukanta^15^, Michael Boehnke^2^, Heikki A. Koistinen^11,16,17^, Markku Laakso^18,19^, Mario Falchi^1^, Jordana T. Bell^1^, Laura J. Scott^2**^, Karen L. Mohlke^3**^ and Kerrin S. Small^1**^

**Table of contents**

Supplementary notes …………………………………………………………………........ 3

- S1: Expression of *ACE* and *TMPRSS2* across TwinsUK tissues …………..… 3
- S2: Assessment of influence of genetic and epigenetic factors on adipose tissue *ACE2* expression …………………………………………………………... 4
- S3: Association of adipose tissue *ACE2* expression with gene expression levels of other key components of the renin-angiotensin system (RAAS) and inflammatory markers …………………………………………………………...... 5

Supplementary materials and methods …………………………………………………. 6

Supplementary Figure 1 …………………………………………………………………. 10

Supplementary Figure 2 …………………………………………………………………. 11

Supplementary Figure 3 …………………………………………………………………. 12

Supplementary Figure 4 …………………………………………………………………. 13

Supplementary Figure 5 …………………………………………………………………. 14

Supplementary Figure 6 …………………………………………………………………. 15

Supplementary Figure 7 …………………………………………………………………. 16

Supplementary Figure 8 …………………………………………………………………. 18

Supplementary Figure 9 …………………………………………………………………. 19

Supplementary references ………………………………………………………………. 20

**Supplementary Notes**

**S1: Expression of *ACE* and *TMPRSS2* across TwinsUK tissues**

We also assessed expression of *ACE2* in skin, lymphoblastoid cell lines (LCLs) and whole blood in TwinsUK. In matched samples from the same subjects in TwinsUK, *ACE2* expression was lower in skin than in adipose tissue (Supplementary Figure 3), and undetectable in lymphoblastoid cell lines and whole blood.

Beyond *ACE2*, two other components of the RAAS system of interest are angiotensin-converting enzyme (*ACE*), and transmembrane protease, serine 2 (*TMPRSS2*). ACE plays a central role in regulation of blood pressure^18^, while TMPRSS2 has received attention since its identification as a cofactor aiding viral invasion by SARS-CoV-2^19^. We therefore explored their expression and correlation patterns in the TwinsUK multi-tissue sample. *ACE* expression was detected in all four tissues, while *TMPRSS2* expression passed our filters only in skin. When comparing normalised, non-transformed gene counts per million (TMM-adjusted CPMs; TMMAdjCPMs), *ACE* had higher expression levels than *ACE2*, with expression much lower in LCLs and whole blood compared to adipose tissue and skin (median[SD] *ACE* adipose = 41.65[14.02]; median[SD] *ACE* skin = 33.30[16.50]; median[SD] *ACE* LCL = 0.17[0.23]; median[SD] *ACE* whole blood = 3.53[1.70]) (Supplementary Figure 4). *TMPRSS2* expression in skin was also higher than that of *ACE2* (median [SD] *TMPRSS2* skin = 18.63[16.42]). We observed an inverse correlation between *ACE2* and *ACE* expression in adipose tissue (Spearman Rho = -0.19; *P* = 8.40 x10^-5^) but not skin (Spearman Rho = -0.03; *P* = 0.53). No correlation was observed between *ACE2* expression in adipose tissue and skin in the same subjects (Spearman Rho = -0.06; *P* = 0.23). On the other hand, *ACE* expression in adipose tissue and skin was positively correlated (Spearman Rho = 0.27; *P* = 2.21 x10^-7^), with no significant correlation across the remaining tissues (Supplementary Figure 4).

**S2: Assessment of influence of genetic and epigenetic factors on adipose tissue *ACE2* expression**

Genetic factors may also influence risk of severe COVID-19^16^. Using the twin structure of TwinsUK, we estimated the heritability of adipose tissue *ACE2* expression to be 0.32 [95% CI = 0.14-0.50] (Supplementary Figure 7). To identify proximal genetic variants associated with *ACE2* expression, we conducted a cis-eQTL meta-analysis of all three studies, including 1,151 participants for whom chromosome X genotype data were available (See Supplementary Methods section). No genetic variants within 1Mb of *ACE2* were associated with adipose tissue *ACE2* expression after correction for multiple testing (minimum *P* = 2.58x10^-3^ at chrX:15263126_A/C) (Supplementary Figure 7 and Supplementary Table 9). This result was consistent with the absence of an *ACE2* cis-eQTL signal in numerous GTEx tissues^17^. We also assessed whether any trans-eQTL variants genome-wide were associated with *ACE2* expression levels; no variants were associated with *ACE2* expression at genome-wide significance (Supplementary Figure 7 and Supplementary Table 10). Neither adjustment for MVEC proportion nor meta-analysis of males and females separately identified significant cis- or trans-eQTLs in our analyses.

Finally, we explored epigenetic effects on *ACE2* expression using matched adipose tissue methylation data, measured on the Illumina Infinium HumanMethylation450 BeadChip for 540 participants in TwinsUK^13^. Similar to our eQTL results, no proximal methylation probes were associated with genetic variants in cis or trans. In addition, no proximal methylation probes were associated with adipose *ACE2* expression (minimum *P* = 0.048 at cg08559914), indicating that methylation at measured sites in our sample does not appear to be a reliable proxy for adipose tissue gene expression at this locus (Supplementary Figure 8).

**S3: Association of adipose tissue *ACE2* expression with gene expression levels of other key components of the renin-angiotensin system (RAAS) and inflammatory markers**

To further investigate the links between adipose tissue *ACE2* expression and inflammation, we also sought to investigate whether we could observe associations at the transcriptional level between other key components of the RAAS, or inflammatory markers, and expression of *ACE2*. We found lower *ACE2* expression to be associated with higher expression of *CD68* (β [95% CI] = -0.14 [0.25;-0.04]; *P* = 5.65x10^-3^), a macrophage marker in adipose tissue. No other transcriptional associations between *ACE2* expression and any of *AGT*, *AGTR1*, *CCL2*, *IL1B*, *TNF*, or *IL6* met the threshold for significance after correction for multiple testing (MTC threshold: *P* < 7.0x10^-3^) (Supplementary Table 8).

**Supplementary materials and methods**

***ACE2* heritability analyses**

Heritability of gene expression levels of *ACE2* was assessed in the TwinsUK sample using an ACE model to decompose variance in gene expression residuals, adjusted for technical covariates, into additive genetic effects (A), common environmental effects shared by both mono- and dizygotic co-twins (C) and unique environmental influences (E). ACE models were fitted using the twinlm function from the mets package^1,2^ in R version 3.5.1^3^. Heritability models included age as a covariate.

**Genotype data, imputation and quality control**

In TwinsUK, imputed genotype data and adipose tissue gene expression data were available for 722 females. TwinsUK, METSIM and FUSION genotype data were generated as previously described^4,5,6^, and were imputed to the Haplotype Reference Consortium panel (HRC version 1.1). Variants with MAF < 0.01, imputation R^2^ < 0.5, and Hardy Weinberg *P* < 1 x 10^-6^ were excluded. HRC-imputed genotype data were used for genome-wide association analysis of *ACE2* expression for all studies.

For *ACE2* ciseQTL analyses, in TwinsUK, HRC-imputed genotypes (HRC version 1.1) were available only for autosomes. Chromosome X genotypes were called from low depth 7x sequencing as part of the UK10K project, as previously described^7^ and were available for 490 of 765 TwinsUK participants for whom adipose tissue gene expression data were available. 39 participants were excluded from cis-eQTL analyses due to skewing of X chromosome inactivation^8^. In METSIM and FUSION, HRC-imputed genotypes (HRC version 1.1) were available for all chromosomes including the X in all participants^5,6^. In males, chromosome X variants were coded as 0,1 alleles.

**Cis-eQTL analyses of adipose tissue *ACE2* expression**

We defined proximal genetic variants to be those within 1Mb on either side of the *ACE2* transcription start site. For TwinsUK, we adjusted gene counts for family structure using a linear mixed effects model including family and zygosity as random effects. In each study, cis-eQTL analyses were conducted using QTLTools, including BMI and 40 PEER factors^9^ as covariates.

**Genome-wide association analysis of *ACE2* expression**

We conducted a GWAS of *ACE2* expression, to identify trans-eQTLs that may modulate *ACE2* expression. All variants greater than 1Mb from the *ACE2* transcription start site were included in this analysis. For TwinsUK, *ACE2* rank inverse normalised gene counts were first adjusted for family structure and technical covariates using a linear mixed effects model including mean GC content and median insert size as fixed effects and sample processing date, primer index, RNA extraction batch, family and zygosity as random effects. GWAS of *ACE2* expression rank inverse normalised expression (METSIM and FUSION) or residuals as described above (TwinsUK) was then conducted using QTLTools^10^ for all studies. Covariates included age, BMI, sample median TIN^11^ and genotyping chip as covariates (TwinsUK). For METSIM, covariates included age, BMI, read deletion size, mean read insertion size, sample median TIN, sequencing batch, and blood proportion as covariates. For FUSION, covariates included were sex, age, BMI, RIN, median TIN, collection site, batch, four genetic PCs, mean GC content, and median insert size as covariates. FUSION also performed sex-stratified GWAS using the same covariates except sex.

**Meta-analysis of *ACE2* expression cis-eQTL and GWAS association results**

Cis-eQTL and genome-wide association analyses of adipose tissue *ACE2* expression from the TwinsUK, METSIM and FUSION cohorts were meta-analysed using the sample-size based method implemented in METAL^12^. Meta-analysis was limited to variants present in a minimum of two out of the three studies (cis-eQTL: N_subjects_ = 1,157, N_variants_ = 2,669 variants; *ACE2* expression GWAS: N_subjects_ = 1,428, N_variants_ = 7,652,879 variants). As the *ACE2* gene is located on the X chromosome, we conducted meta-analyses including all participants, as well as sex-stratified analyses.

The threshold for significance of association of cis-eQTL variants with *ACE2* expression was *P* < 6.94 x 10^-5^, calculated using the EigenMT approach to estimate the number of independent tests in the region in the largest sample (TwinsUK). 721 principal components were found to account for > 99% of the variance in the variants tested. For the identification of trans-eQTL variants associating with *ACE2* expression, a genome-wide threshold of significance of *P* < 5.0 x 10^-8^ was used.

***ACE2* methylation analyses in TwinsUK**

DNA methylation patterns were profiled with the Illumina Infinium HumanMethylation450 BeadChip for 540 adipose tissue biopsy samples, as previously described^13^. Methylation β-values were pre-processed with the ENmix package^14^ within the R environment and inverse-normalised. After filtering out samples skewed for X-chromosome inactivation, there were 495 samples for downstream analyses. For GWAS of DNA methylation levels in CpGs annotated within the *ACE2* gene (i.e., methylation quantitative trait loci, meQTLs) DNA methylation values were first adjusted for covariates in a linear mixed-effects model with fixed effect factors including smoking, age and bisulfite conversion efficiency, and zygosity, and random effects including position on array and family. MeQTL analysis was then performed on the resulting residuals using Matrix eQTL^15^ in both cis (2Mb window, with a total of 325 samples with available genotypes) and in trans for all variants greater than 1Mb either side of the CpGs annotated within the *ACE2* gene. The association between *ACE2* expression and proximal methylation probes was carried out in Matrix eQTL, using methylation and *ACE2* gene expression residuals adjusted for technical and family confounders as previously described (as well as smoking status for methylation data). The methylation-expression association models included age, BMI, T2D status, and MVEC estimation as covariates.

**Supplementary Figures**

**Supplementary Figure 1:** **Gene expression levels of *ACE2* across 54 tissues in the GTEx resource.** Expression levels plotted as log10(TPM+1).

**Supplementary Figure 2: Age and BMI distributions in the TwinsUK, METSIM and FUSION studies.** a-c) Age distribution in TwinsUK, METSIM and FUSION studies. d-f) BMI distribution in TwinsUK, METSIM and FUSION studies. Plots include only non-diabetic subjects.

**Supplementary Figure 3: *ACE2* expression levels across studies and tissues.** *ACE2* expression is plotted as TMM-adjusted counts per million. a) TwinsUK adipose tissue b) METSIM study adipose tissue c) FUSION study adipose tissue d) TwinsUK skin.

**Supplementary Figure 4: Expression levels of RAS genes *ACE* and *TMPRSS2* across tissues in TwinsUK**. a-e) Histograms of expression levels of ACE and TMPRSS2 expression in TwinsUK skin, LCL whole blood, and adipose tissue, as indicated. f) Correlation plot of RAAS gene expression across tissues in unrelated subjects from the TwinsUK study (n=463 participants).

**Supplementary Figure 5: *ACE2* expression in adipose tissue and skin show inverse direction of association with age**. a) TwinsUK adipose tissue. b) TwinsUK skin. Each point represents an individual. Participant age was rank inverse-normal transformed. *ACE2* expression residuals plotted were adjusted for technical covariates and BMI to correspond to the reported association model.

**Supplementary Figure 6: Correlation plot of adipose tissue *ACE2* expression, phenotypic variables, and adipose tissue estimated cell type compositions in TwinsUK.** Adipose tissue *ACE2* expression residuals were adjusted for RNASeq technical covariates**.** Correlations were assessed in unrelated subjects (n=441).

**Supplementary Figure 7: Genetic factors influencing adipose tissue expression of *ACE2*.** a) Heritability of *ACE2* expression, assessed using an ACE model in the TwinsUK study. *ACE2* expression was partitioned into that which could be explained by (A) additive genetic effects (h^2^), common environment (C) and unique environment (E). Error bars show the 95% confidence intervals. b) Regional plot of *ACE2* cis-eQTL associations in 1,157 participants, within 1Mb either side of the *ACE2* transcription start site. c) Manhattan plot of genome-wide association study meta-analysis of adipose tissue *ACE2* expression in 1,428 participants. For plots b-c, the x-axis denotes chromosomal position, and the y-axis -log10(P-value). The red line indicates the threshold for genome-wide significance (P < 5x10^-8^).

**Supplementary Figure 8: Adipose tissue DNA methylation at methylation probes proximal to the *ACE2* gene is not associated with *ACE2* expression levels.** Each point represents a single subject, with adipose tissue methylation beta values plotted on the x-axis and adipose tissue *ACE2* expression on the y-axis. Rank-based inverse normal transformation was applied to both methylation and gene expression data.

**Supplementary Figure 9: Estimated cell type proportion of whole blood in the METSIM and FUSION studies.** Cell type proportions were estimated using CIBERSORT (METSIM) and DESeq2 (FUSION) (See methods).

**Supplementary references**

1 Scheike TH, Holst KK, Hjelmborg JB. Estimating heritability for cause specific mortality based on twin studies. *Lifetime Data Anal* 2014; **20**: 210–233.

2 Holst KK, Scheike TH, Hjelmborg JB. The liability threshold model for censored twin data. *Comput Stat Data Anal* 2016; **93**: 324–335.

3 R Development Core Team. R: A Language and Environment for Statistical Computing. *R Found Stat Comput Vienna Austria* 2013; **0**: {ISBN} 3-900051-07-0.

4 Hysi PG, Young TL, MacKey DA, Andrew T, Fernández-Medarde A, Solouki AM *et al.* A genome-wide association study for myopia and refractive error identifies a susceptibility locus at 15q25. *Nat Genet* 2010; **42**: 902–905.

5 Civelek M, Wu Y, Pan C, Raulerson CK, Ko A, He A *et al.* Genetic Regulation of Adipose Gene Expression and Cardio-Metabolic Traits. *Am J Hum Genet* 2017; **100**: 428–443.

6 Leland Taylor D, Jackson AU, Narisu N, Hemani G, Erdos MR, Chines PS *et al.* Integrative analysis of gene expression, DNA methylation, physiological traits, and genetic variation in human skeletal muscle. *Proc Natl Acad Sci U S A* 2019; **166**: 10883–10888.

7 Walter K, Min JL, Huang J, Crooks L, Memari Y, McCarthy S *et al.* The UK10K project identifies rare variants in health and disease. *Nature* 2015; **526**: 82–89.

8 Zito A, Davies MN, Tsai P-C, Roberts S, Andres-Ejarque R, Nardone S *et al.* Heritability of skewed X-inactivation in female twins is tissue-specific and associated with age. doi:10.1038/s41467-019-13340-w.

9 Stegle O, Parts L, Piipari M, Winn J, Durbin R. Using probabilistic estimation of expression residuals (PEER) to obtain increased power and interpretability of gene expression analyses. *Nat Protoc* 2012; **7**: 500–7.

10 Delaneau O, Ongen H, Brown AA, Fort A, Panousis NI, Dermitzakis ET. A complete tool set for molecular QTL discovery and analysis. *Nat Commun* 2017; **8**: 15452.

11 Wang L, Wang S, Li W, Li Y, Eckel-Passow JE, Dasari S *et al.* RSeQC: quality control of RNA-seq experiments. *Bioinformatics* 2012; **28**: 2184–2185.

12 Willer CJ, Li Y, Abecasis GR. METAL: fast and efficient meta-analysis of genomewide association scans. *Bioinforma Appl NOTE* 2010; **26**: 2190–2191.

13 Grundberg E, Meduri E, Sandling JK, Hedman ÅK, Keildson S, Buil A *et al.* Global analysis of dna methylation variation in adipose tissue from twins reveals links to disease-associated variants in distal regulatory elements. *Am J Hum Genet* 2013. doi:10.1016/j.ajhg.2013.10.004.

14 Xu Z, Niu L, Li L, Taylor JA. ENmix: A novel background correction method for Illumina HumanMethylation450 BeadChip. *Nucleic Acids Res* 2016. doi:10.1093/nar/gkv907.

15 Shabalin AA. Matrix eQTL: ultra fast eQTL analysis via large matrix operations. *Bioinformatics* 2012; **28**: 1353–8.

16 The COVID-19 Host Genetics Initiative, a global initiative to elucidate the role of host genetic factors in susceptibility and severity of the SARS-CoV-2 virus pandemic. *Eur J Hum Genet* 2020; **28**: 715–718.

17 He Y. Tissue specific expression and genetic regulation of SARS-CoV-2 receptors ACE2 and TMPRSS2. https://medium.com/@yuanhe716/tissue-specific-expression-and-genetic-regulation-of-sars-cov-2-receptors-ace2-and-tmprss2-d75d76bf45fb (accessed 13 Jun2020).

18 SKEGGS LT, KAHN JR, SHUMWAY NP. The preparation and function of the hypertensin-converting enzyme. *J Exp Med* 1956; **103**: 295–299.

19 Hoffmann M, Kleine-Weber H, Schroeder S, Krüger N, Herrler T, Erichsen S *et al.* SARS-CoV-2 Cell Entry Depends on ACE2 and TMPRSS2 and Is Blocked by a Clinically Proven Protease Inhibitor. *Cell* 2020; **181**: 271-280.e8.
